# A 3D model to evaluate retinal nerve fiber layer thickness deviations caused by the displacement of optical coherence tomography circular scans in cynomolgus monkeys (*Macaca fascicularis*)

**DOI:** 10.1371/journal.pone.0237858

**Published:** 2020-08-21

**Authors:** Stephanie Niklaus, Pascal W. Hasler, Timothy Bryant, Sébastien Desgent, Mark Vezina, Tobias K. Schnitzer, Peter M. Maloca, Nora Denk

**Affiliations:** 1 Pharma Research and Early Development (pRED), Pharmaceutical Sciences (PS), Roche Innovation Center Basel, Basel, Switzerland; 2 OCTlab, Department of Ophthalmology, University Hospital Basel, Basel, Switzerland; 3 Department of Ophthalmology, University of Basel, Basel, Switzerland; 4 Charles River Laboratories (Montreal), Senneville, Québec, Canada; 5 Institute of Molecular and Clinical Ophthalmology Basel (IOB), Basel, Switzerland; 6 Moorfields Eye Hospital, London, United Kingdom; University of Florida, UNITED STATES

## Abstract

The main objective of the study was to analyze deviations in retinal nerve fiber layer (RNFL) thickness measurements caused by the displacement of circular optic disc optical coherence tomography scans. High-density radial scans of the optic nerve heads of cynomolgus monkeys were acquired. The retinal nerve fiber layer was manually segmented, and a surface plot of the discrete coordinates was generated. From this plot, the RNFL thicknesses were calculated and compared between accurately centered and intentionally displaced circle scans. Circle scan displacement caused circumpapillary retinal nerve fiber layer thickness deviations of increasing magnitude with increasing center offset. As opposed to the human eye, horizontal displacement resulted in larger RNFL thickness deviations than vertical displacement in cynomolgus monkeys. Acquisition of high-density radial scans allowed for the mathematical reconstruction and modelling of the nerve fiber layer and extrapolation of its thickness. Accurate and strictly repeatable circle scan placement is critical to obtain reproducible values, which is essential for longitudinal studies.

## Introduction

Optical coherence tomography (OCT) has emerged as the primary modality to image ocular structures [1, 2]. Using low coherence interferometry, OCT generates high-resolution cross-sectional images by capturing optical scattering from the tissue and thereby depicting retinal layers, the optic disc, and beyond. In preclinical ophthalmology toxicology studies, OCT has become an indispensable imaging tool enabling non-invasive real-time observation of the retina and optic nerve during the time-course of the study [3, 4]. Cynomolgus monkeys are frequently used for safety profiling of new drug candidates. Their anatomical similarity to humans, including their congruent eye structure with the presence of the macula and the relatively large eye size compared to other research animals such as rodents renders cynomolgus monkeys suitable model organisms for the safety assessment of ocular compounds during which ocular structures are monitored by OCT [3–5].

OCT enabled the detection of glaucoma years before the onset of visual field defects and therefore the analysis of the peripapillary retinal nerve fiber layer (RNFL) is used as an important biomarker to diagnose and monitor glaucoma [6, 7]. Additionally, RNFL assessment is routinely performed in preclinical settings, to monitor the integrity of the optic nerve and peripapillary retina in monkeys. The RNFL is typically analyzed using a 3.4 mm diameter circular scan centered on the optic disc and represented in temporal superior nasal inferior temporal (TSNIT) plots [8]. In comparison to other circular scan diameters, a 3.4 mm diameter has been shown to be superior in the reproducibility of RNFL thickness results, thus leading to higher levels of inter- and intrasession reproducibility in humans [9, 10]. Further, this proposed diameter has been shown beneficial to reliably diagnose glaucoma [11–14]. Various 3.4 mm optic disc scan pattern have been successfully described [9, 15–20]. The importance of accurate centering of the optic disc circle scan to obtain reliable and reproducible RNFL measurements was demonstrated in humans. Small circle scan displacements by 0.1 mm have been shown to result in remarkable RNFL thickness profile deviations so that such erroneous measurements could also have a clinical impact [21, 22]. However, there are currently no available data to show how circle scan misplacement impacts RNFL thickness in cynomolgus monkeys. Therefore, interpretations of RNFL thickness changes in the course of a toxicity study were difficult as differentiation of actual pathological changes from misplacement artefacts can be challenging

Thus, a proposed three-dimensional (3D) model of the RNFL was developed in cynomolgus monkeys to evaluate for RNFL deviations in animals.

## Material and methods

### Animals and husbandry

Data for this study were acquired during the baseline examination of a routine pharmaceutical product development study and thus no additional animals were used. Animal care and experimentation were conducted in accordance with the Association for Assessment and Accreditation of Laboratory Animal Care (AAALAC) and the Canadian Council on Animal Care (CCAC) guidelines. The protocol has been reviewed and approved by the Institutional Animal Care and Use Committee (CRL Montreal IACUC).

Three healthy, treatment-naïve Mauritian cynomolgus monkeys, *Macaca fascicularis* (2 female and 1 male) who were between 30 and 50 months of age (body weight range = 2.5–5.5 kg) were included in the study. For 3R reasons, the reduced number of three animals was chosen for this feasibility study. All three animals were tested negative for tuberculosis. Animals were kept in groups of three animals at temperatures between 20°C and 26°C (humidity = 30–70%) and maintained on a 12 h light/dark cycle in stainless steel cages, according to the European housing standards as described in the Annex III of the Directive 2010/63/EU. Animals were provided with PMI Nutrition International Certified Primate Chow and tap water treated by reverse osmosis and ultraviolet irradiation. Environmental and psychological enrichment was always provided to animals, except during study procedures.

### Optical coherence tomography

For OCT scan acquisition, animals were anesthetized with an intra-muscular (IM) injection of a mixture of 10 mg/kg ketamine and 25 μg/kg dexmedetomidine. Prior to imaging, the animal’s pupils were dilated with topical tropicamide drops and the eyes were stabilized at a central position by the IM administration of 0.2 mg/kg midazolam. This anesthesia protocol was well suited in regards of length of anesthesia adapted to imaging duration, the ability to keep the eyes centrally positioned, the ease of use as well as safety and tolerability for the animals. Spectral-domain OCT (SD-OCT) scans were acquired with the Heidelberg Spectralis HRA+OCT platform (Heidelberg Engineering, Heidelberg, Germany). High-density radial scans (consisting of 96 b-scans) positioned in the center of the optic nerve head (ONH) were acquired averaging 15–20 images per scan using the Automatic Real-time Tracking (ART) settings.

### Image processing and mathematical modelling

**Fig 1:**
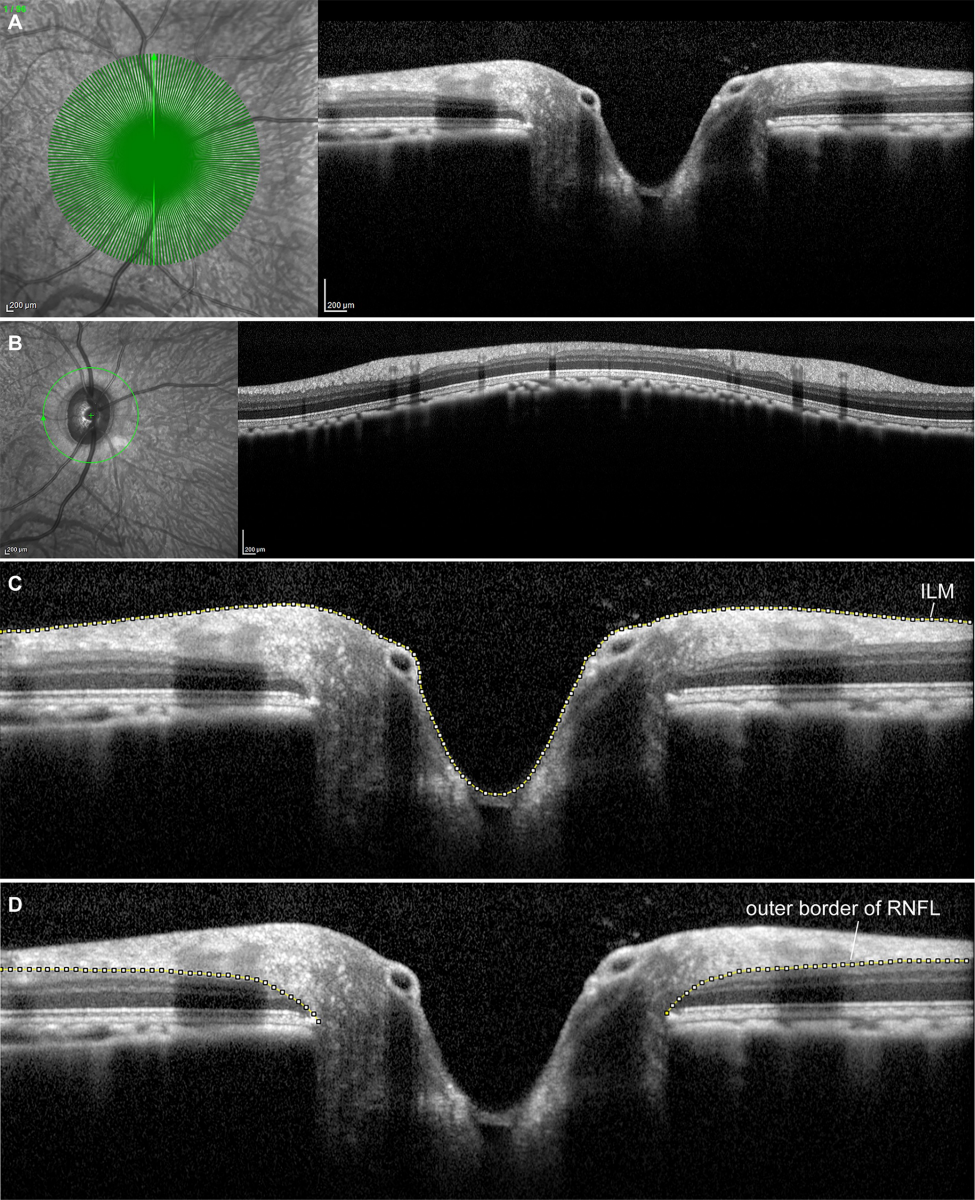
Optical Coherence Tomography (OCT) scans used for mathematical modelling of the RNFL. (A) Radial scans consisting of 96 b-scans (green lines) that were acquired for modelling of the RNFL. The OCT b-scan (right) corresponds to the highlighted green b-scan shown on the infrared fundus image (left) (B) Example of a circumpapillary circle scan. (C) Manual segmentation of the inner limiting membrane (ILM) and the outer border of the retinal nerve fiber layer (RNFL) (D) to generate the three-dimensional model of the RNFL surface.

Mathematical modelling was applied to construct the RNFL surface from discrete data points obtained by the high-density radial scans of the right eye of each subject: The inner limiting membrane (ILM) and outer border of the RNFL were manually delineated in all 96 b-scans per radial scan using Fiji, an open-source platform for biological-image analysis [23] (Fig 1). Segmentation lines were interpolated every 10 pixels and the resulting discrete X-Y coordinates of the interpolated values were exported. The Y-coordinates of the b-scans were used to compose the Z values of the 3D model. Sine and cosine functions were applied to obtain the X and Y coordinates of the 3D model from the b-scan X coordinates. The X and Y coordinates refer to the following directions: X positive = nasal; X negative = temporal; Y positive = superior; Y negative = inferior.

A 3D surface plot of the ILM and the outer border of the RNFL was generated from the discrete X, Y, and Z values using Delaunay triangulation with MATLAB (R2017b, Mathworks, Natick, United States). Peripapillary RNFL thickness values were obtained by plotting a cylinder (r = 1.7 mm) and extracting the interpolated distance between the ILM-cylinder and the outer border of the RNFL- cylinder intersection (see full Matlab code in S1 Code).

Peripapillary RNFL thickness profiles along the virtual circle scan were extracted for well-centered (baseline) and misplaced circles. Virtual scan circles were displaced from the center of the ONH in four directions (temporal, superior, nasal, and inferior) at different distances from the disc center (0.074 mm, 0.1 mm, 0.2 mm, 0.3 mm, 0.4 mm, and 0.5 mm) to also allow comparison of scan displacement effect between humans and cynomolgus monkeys with difefernt eye size and different axial length. Circumpapillary circles were evenly divided into 10 segments (S1 to S10, clockwise direction), with S1 being the virtual start of a circular scan (temporal, 9 o’clock position). The average percentage of RNFL thickness changes between misplaced and baseline scans was calculated for each segment. RNFL thickness profiles are shown in TSNIT plots. Peak thicknesses in TSNIT plots are defined as maximum RNFL thickness along the circle scan; one peak is found in the superior and one in the inferior quadrant. The peak distance in TSNIT plots is defined as the distance between the two thickness peaks relative to the nasal position.

RNFL thickness profile plots (TSNIT plots) and heat maps showing the percentage of RNFL thickness changes caused by misplacement were generated using GraphPad Prism (Version 7.04, San Diego, United States). All images were assembled using Adobe Photoshop CC 2018 and Adobe Illustrator CC 2018.

## Results

### Mathematical RNFL model

**Fig 2:**
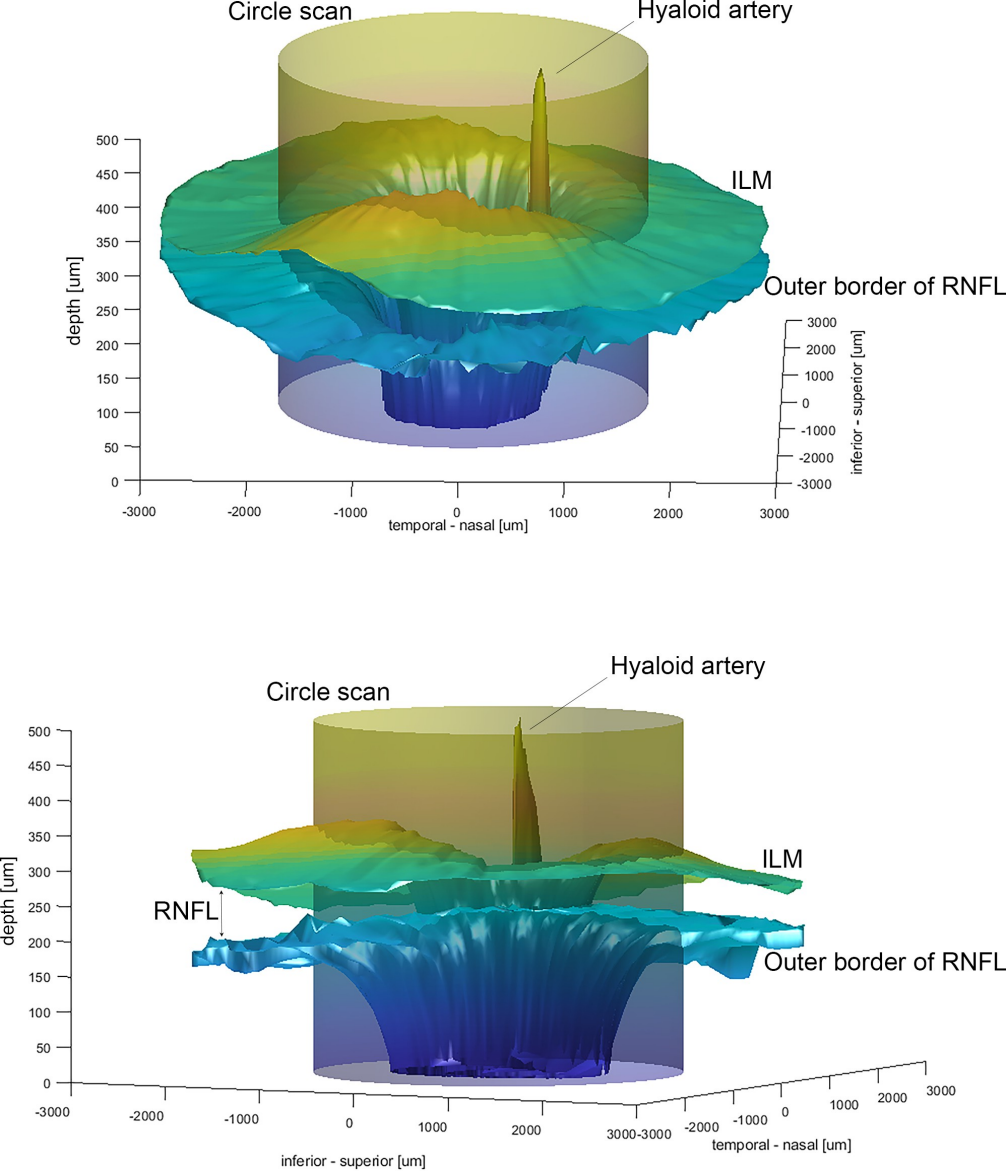
Three-dimensional model of the ILM and the outer border of the surface of the RNFL. The upper and lower panels show the three-dimensional (3D) computation of the inner limiting membrane (ILM) and the outer border of the retinal nerve fiber layer (RNFL) from different point of views. The 3.4 mm diameter of the cylinder mimics a circular scan. The distance between the two surfaces along the cylinder equals the circumpapillary RNFL thickness.

The 3D RNFL surface plot generated from discrete interpolated data points (see S1, S2 and S3 Tables for RNFL coordinates) of the segmented high-density OCT radial scan showed high inter-subject similarity in circumpapillary RNFL thickness (Fig 1) and is displayed in Fig 2. Similar to the human ONH, the RNFL showed increased thickness in the superior and inferior quadrants. Furthermore, the model clearly displayed an example of a Bergmeister’s papilla, by the presence of remnants of the fetal hyaloid artery (Fig 2).

**Fig 3:**
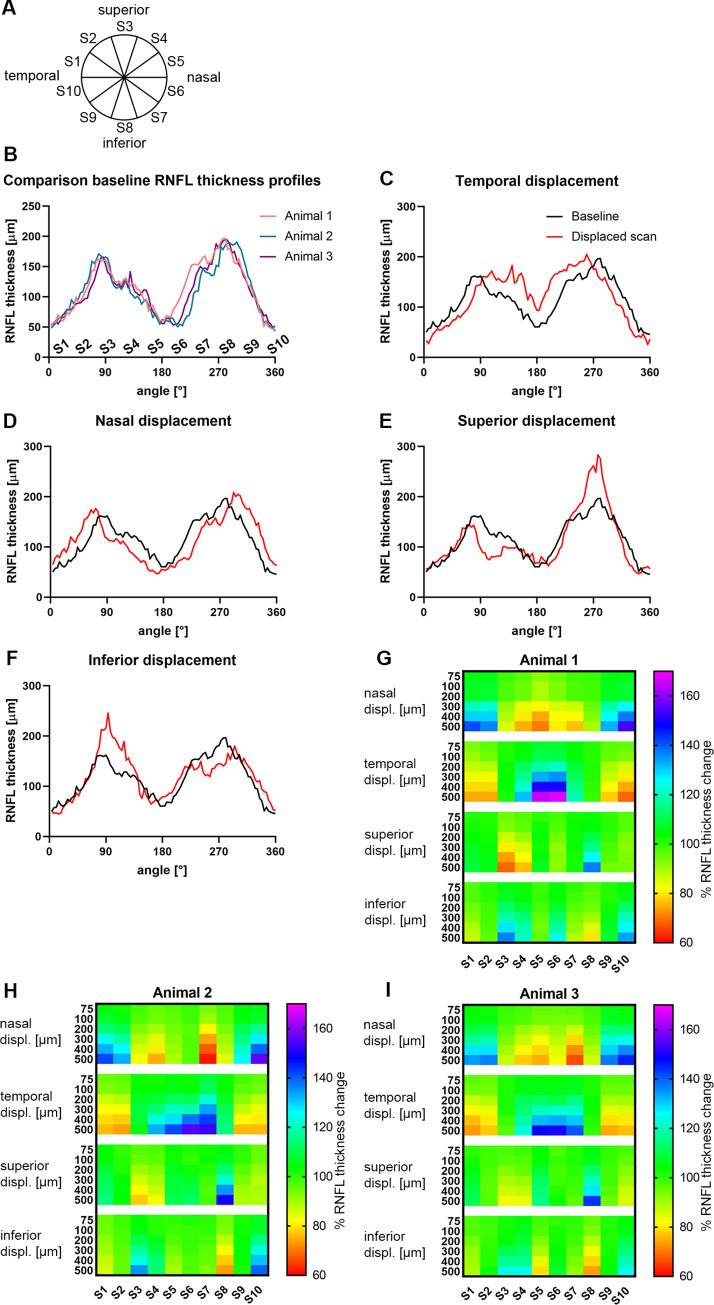
RNFL thickness deviations caused by displacements of the scan circle. (A) Circle scans were divided into 10 equal segments (S 1–10) in a clockwise manner, with S1 marking the start of the scan at the 9 o’clock position (temporal). (B) A temporal superior nasal inferior temporal plot showing a high degree of similarity in circumpapillary retinal nerve fiber layer (RNFL) thickness between the three subjects. (C-F) RNFL thicknesses of a well-centered circle scan before (black line) and after 0.5 mm displacement (red line) into temporal (C), nasal (D), superior (E), and inferior (F) directions. (G-H) Heat map display of the RNFL thickness changes of the three subjects (G = animal 1; H = animal 2; I = animal 3) after 0.074 mm, 0.1 mm, 0.2 mm, 0.3 mm, 0.4 mm, and 0.5 mm circle displacements in the nasal, temporal, superior, and inferior directions. displ. = displacement.

The circumpapillary RNFL thickness profiles computed from the model along a virtual 3.4 mm circle were compared between the three study subjects (animals 1 to 3) and plotted in Fig 3. The three monkeys generally showed high similarity in RNFL thickness, with remarkable inter-subject similarities in Sectors 1 to 5 and 8 to 10 (S1 to S5 and S8 to S10) (Fig 3B). The inferonasal quadrant (S6 to S7) showed slightly higher between-subject variability in RNFL thickness (Fig 3B; not quantified).

### Effect of circle scan displacement on circumpapillary RNFL thickness profiles

The virtual scan circle RNFL thickness profiles of well-centered (baseline) and displaced scans were compared. Fig 3C–3F shows how the thickness profiles changed with respect to 0.5 mm displacements of the scan center in four different directions (i.e., displacement in the temporal (Fig 3C), nasal (Fig 3D), superior (Fig 3E), and inferior (Fig 3F) directions). These plots show that horizontal displacement (nasal and temporal displacement) affects peak distance but has no significant impact on RNFL peak thickness values. However, vertical displacement affects the peak thickness values, with superior displacement causing increased thickness values of the inferior peak (Fig 3E) and inferior displacement evoking an increase in superior peak thickness (Fig 3F).

Analysis of thickness measurement changes in dependency of displacement distance show that RNFL thickness measurement errors increase with increasing offset, independent of displacement direction. Small circle displacements of 0.074 mm cause RNFL thickness changes of up to 9.7% and displacements of 0.1 mm evoke RNFL thickness deviations of up to 11% (per segment, per animal). As the distance from the disc center increases, the RNFL measurement errors increase reaching values of up to a 67% increase in thickness in the nasal region (S5 and S6) after a 0.5 mm displacement in the temporal direction and a 39% decrease in thickness in S7 (inferior-nasal) after a nasal displacement of 0.5 mm (Fig 3G–3I).

Each of the four displacement directions (temporal, superior, nasal, and inferior) elicits a unique RNFL thickness error profile that is similar in all three subjects. Horizontal misplacement evokes errors in the temporal and nasal segments, but only has minor effects in the superior and inferior segments. More specifically, scan displacement towards the nasal direction causes an increase in temporal and a decrease in nasal RNFL thickness whereas temporal displacement causes an increase and decrease in the nasal and temporal segments, respectively. Contrary to horizontal displacements, vertical displacements mainly evoke RNFL thickness changes in the superior and inferior segments. Additionally, small errors are induced in the nasal segment by vertical displacement, however, temporal values remain virtually unaffected. Scan displacement in the superior direction results in increased RNFL thickness in the inferior segment (S8) and decreased thickness in the superior segments (S3 and S4). Scan displacement in the inferior direction causes an increase in RNFL thickness in the superior segments (S2, 3) and a decrease in the inferior segment (S8).

## Discussion

Retinal nerve fiber layer measurements have been shown to be a crucial biomarker for glaucoma in humans [24]. However, displacement of the optic disc circle scan causes erroneous RNFL thickness values in humans which may lead to false diagnosis or conclusions. Vertical shifts (superior and inferior directions) were reported to affect RNFL thickness profiles more than horizontal shifts (nasal and temporal) in humans. Additionally, the RNFL thicknesses of the temporal and nasal quadrants were found to be more robust against misplacement errors compared to the superior and inferior quadrants [21, 22]. These findings emphasized the importance of acquiring well-centered scans to correctly obtain and interpret RNFL results. While in human subjects advances in OCT technology support precise alignment of scans using anatomical landmarks, such as the Anatomic Positioning System (APS) by Heidelberg Spectralis, such technology has to our knowledge not yet been validated for use in non-human species and is therefore currently not standardly used in preclinical studies.

In this context, this study evaluated successfully a novel RNFL assessment by providing a mathematical 3D reconstruction method of the RNFL distribution to appreciate such potential errors also in animals.

### RNFL profiles of cynomolgus monkeys

The 3D surface plots show a generally high level of similarity between the cynomolgus monkey and human ONHs with regard to the superior and inferior RNFL thickness peaks as reported before [25]. Additionally, the model clearly depicted the remnants of the fetal hyaloid artery. The age of complete disappearance of the hyaloid artery varies significantly in monkeys, and therefore the presence of remnants (known as Bergmeister’s papilla) are often present up to adulthood [26] and can cause segmentation artifacts.

Circumpapillary RNFL thicknesses of the well-centered and virtually displaced circle scans were computed from the 3D model and compared in the three study subjects. This comparison shows remarkable similarity in circumpapillary RNFL thickness between the three subjects. In addition, scan circle misplacements caused homogeneous RNFL thickness changes in the monkeys, which further demonstrates the low inter-subject variability in the nerve fiber layer surrounding the optic disc of healthy, treatment-naïve cynomolgus monkeys. Back in the early days of OCT, the scan circle diameter was arbitrarily set to 3.4 mm, based on a study that showed superior reproducibility of a 3.4 mm scan circle compared to smaller diameters and a superior intraclass correlation compared to smaller and larger diameters in healthy humans [10]. Thus, a 3.4 mm diameter has become standard for scan and measurement circles. Low inter-subject RNFL thickness variability in cynomolgus monkeys and similarity in the TSNIT plots between monkeys and humans suggests that 3.4 mm is also suitable for ganglion cell fiber layer analysis in monkeys.

### Direction- and distance-dependent displacement errors

The obtained data show that even in monkeys the centering of the scan is enormously important: Small scan displacements of 0.074 mm caused errors in RNFL thickness values of up to 9.7% (6μm) and displacements of 0.1 mm caused errors in RNFL thickness values of up to 11% per segment. Given the smaller size of the eye of cynomolgus monkeys with an axial length of 74% of the axial length of the human eye [27], we compared a 0.1 mm scan displacements in humans to a 0.074 mm displacement in cynomolgus monkeys. Such a 0.1 mm scan displacement in humans leads to a maximum thickness error in 6.2 μm, similarly a 0.074 mm displacement in cynomolgus monkeys evokes a maximum error of 6 μm. However, given the smaller eye size of the monkey, small displacements evoke larger errors if not normalized to the eye size. The displacement errors in monkeys increase with the offset from the scan center for all 4 directions assessed, similar to humans [21, 22]. Vertical scan displacements resulted in more pronounced deviations in the superior and inferior quadrants than in the nasal and temporal quadrants, whereas horizontal displacements caused more drastic errors in RNFL thickness in the temporal and nasal quadrants, which is all analogous to humans [22]. However, contrary to humans, in cynomolgus monkeys horizontal displacements evoked more drastic deviations than vertical displacements [21, 22], with an up to 67% increase in RNFL thickness (averaged per segment). This 67% increase was detected in the S1 segment (temporal) after a 0.5 mm displacement in the temporal direction, and caused the average segment (S10) RNFL thickness to increase by 56 μm (from 84 μm to 140 μm).

While small displacements (0.074 mm in monkeys and 0.1 mm in humans) result in comparable errors in moneys and human, deviations in cynomolgus monkeys caused by larger displacements by far exceed displacement errors reported in the human eye, where the largest measurement error was found to be 25.9 μm upon 0.5 mm displacement towards inferior direction, which is only roughly 50% of the maximum error in monkeys [21]. Nevertheless, it should be noted that Cheung *et al*. listed measurement errors averaged per quadrant, whereas we list errors averaged per segment (10 segments per circle scan). Summarizing the averages per quadrant was not suitable for monkeys, as displacement errors contained increases and decreases in thickness within quadrants and thus the resulting averages were not meaningful. Although we believe that it is likely, further studies should analyze whether scan displacement actually causes more drastic measurement errors in monkeys, or whether this observation is caused by different segmentation.

This study is limited by the relatively small number of study subjects. Furthermore, the segmentation was performed by only one grader and repeatability of the segmentation was not assessed. In addition, only data from one OCT scanner was processed so that the findings potentially cannot be used interchangeably between different devices. Nevertheless, these important findings derived from a first feasibility study in animals and further studies will investigate the generalizability of the developed method.

## Conclusions

This study highlights the importance of accurate scan circle placement for RNFL thickness analysis in cynomolgus monkeys. Small deviations of the circle center from the disc center cause errors in RNFL thickness, which increase with progressive offset from the disc center. Resulting RNFL thickness errors in cynomolgus monkeys are likely more pronounced compared to humans using the identical displacement directions and distances. Thus, in order to assure reproducibility in longitudinal studies, placement of the circle scan is of utmost importance in order to avoid false interpretations and conclusions. The proposed radial scan pattern and the generated 3D model may aid physicians and researchers in distinguishing pathological thickness profile changes from thickness artifacts caused by misaligned scans.

## Supporting information

S1 Code Matlab code. (PDF)

S1 Table All data points of animal 1. XYZ coordinates, RNFL thickness values of well-centered and misplaced scans and percentage of RNFL thickness change of misplaced scans (averaged per segment S1 to S10).
(XLSX)

S2 Table All data points of animal 2. XYZ coordinates, RNFL thickness values of well-centered and misplaced scans and percentage of RNFL thickness change of misplaced scans (averaged per segment S1 to S10).
(XLSX)

S3 Table All data points of animal 3. XYZ coordinates, RNFL thickness values of well-centered and misplaced scans and percentage of RNFL thickness change of misplaced scans (averaged per segment S1 to S10).
(XLSX)

S1 File E2E animal1. High-density radial scan of animal 1.
(E2E)

S2 File E2E animal2. High-density radial scan of animal 2.
(E2E)

S3 File E2E animal3. High-density radial scan of animal 3.
(E2E)
