# Supplementary material for: A 3D model to evaluate retinal nerve fiber layer thickness deviations caused by the displacement of optical coherence tomography circular scans in cynomolgus monkeys (Macaca fascicularis)
Source: PLoS One. 2020 Aug 21;15(8):e0237858. doi: 10.1371/journal.pone.0237858 (PMC7446827; doi:10.1371/journal.pone.0237858)
Supplement: S1 Code — (PDF) [file pone.0237858.s001.pdf]

Matlab code 3D RNFL model

```
dataset_ilm_um = xlsread('ilm.xlsx');
save('dataset_ilm_um.mat','dataset_ilm_um')

dataset_rnfl_um = xlsread('rnfl.xlsx');
save('dataset_rnfl_um.mat', 'dataset_rnfl_um')

%build a cylinder mimicking the circular scan
r = 1700; %this is the radius of the circular scan in um
t = linspace(0, 2*pi);
%(r*cos(t))+ex --> error x direction. Enter value in equation to move
%cylinder!
%(r*sin(t))+ey --> error y direction. Enter value in equation to move
%cylinder!
cyl = [((r*cos(t))-000); ((r*sin(t))-000)];
cyl1 = repmat(cyl(1,:),100,1);
cyl2 = repmat(cyl(2,:),100,1);
cyl3 = repmat(linspace(-20, 500)',1,100);

%plot cylinder in 3D
figure(1)
surface(cyl1, cyl2, cyl3,'facecolor', [1.0 0.5 0.0], 'EdgeColor', 'none');
alpha 0.5
view(3); camlight; axis vis3d
hold on

%plot surface 1 = ILM
z1 = cyl1
load('dataset_ilm_um.mat')

x1 = dataset_ilm_um(:,1);
y1 = dataset_ilm_um(:,2);
z1 = dataset_ilm_um(:,3);

figure(1)
tri = delaunay(x1,y1);
h = trisurf(tri, x1, y1, z1);

hold on

%plot surface 2 = outer border of RNFL
z2 = cyl2
load('dataset_rnfl_um.mat')

x2 = dataset_rnfl_um(:,1);
y2 = dataset_rnfl_um(:,2);
z2 = dataset_rnfl_um(:,3);

tri = delaunay(x2,y2);

h = trisurf(tri, x2, y2, z2);

axis vis3d
hold on

xlim([-3000,3000])
ylim([-3000,3000])
zlim([0,500])
```

```
axis on
l = light('Position',[-50 15 -150]);

lighting phong
shading interp
colorbar EastOutside
set(gcf, 'Units', 'Normalized', 'OuterPosition', [0, 0.04, 1, 0.96]);



% Set up interpolation function based on scattered data
Interpolant_ilm=scatteredInterpolant(x1, y1, z1);
Interpolant_rnfl=scatteredInterpolant(x2, y2, z2);

% Interpolate on circle coordinates (function evaluation with circle
coordinates as function input)
Intersection_ilm=Interpolant_ilm(cyl(1,:),cyl(2,:));
Intersection_rnfl=Interpolant_rnfl(cyl(1,:),cyl(2,:));

% Calculate distance between intersection on both surfaces
difference=Intersection_ilm-Intersection_rnfl;


figure(42)
subplot(3,1,1)
plot(t,Intersection_ilm)
subplot(3,1,2)
plot(t,Intersection_rnfl)
subplot(3,1,3)
plot(t,difference)

% Difference plot = RNFL thickness
figure(43)
plot(t,difference)
xlabel('angle [Rad]')
ylabel('RNFL thickness [um]')
```
